# Modification of the association between experience of economic distress during the COVID-19 pandemic and behavioral health outcomes by availability of emergency cash reserves: findings from a nationally-representative survey in Thailand

**DOI:** 10.7717/peerj.13307

**Published:** 2022-04-20

**Authors:** Wit Wichaidit, Chayapisika Prommanee, Sasira Choocham, Rassamee Chotipanvithayakul, Sawitri Assanangkornchai

**Affiliations:** 1 Department of Epidemiology, Faculty of Medicine, Prince of Songkla University, Hat Yai, Songkhla, Thailand; 2 Centre for Alcohol Studies, Hat Yai, Songkhla Province, Thailand; 3 Division of Computational Science (Statistics), Faculty of Science, Prince of Songkla University, Hat Yai, Songkhla Province, Thailand

**Keywords:** Emergency savings, Economic distress, Anxiety, Depression, COVID-19

## Abstract

**Background:**

Studies have suggested that economic distress is associated with behavioral health outcomes, while availability of cash reserves for emergencies is associated with a reduction in economic distress. The objective of this study was to assess the extent that the availability of emergency cash reserves modified the association between experience of economic distress during the COVID-19 pandemic and behavioral health outcomes in the general adult population of Thailand.

**Methods:**

We conducted a nationally-representative phone-based survey in late April 2021. Survey questions included questions on experience of economic distress, and a question on what participants would do to cover a 5,000 Thai Bahts (THB) emergency expense within one week, anxiety and depression screening questions, and questions regarding sleep, exercise, gambling, smoking, and drinking behaviors. We analyzed data using descriptive statistics and multivariate logistic regression analyses with adjustment for complex survey designs, and stratified analyses with assessment of heterogeneity of odds ratios between strata and assessment of additive and multiplicative interactions.

**Results:**

A total of 1,555 individuals from 15 provinces participated in the survey (participation rate = 68.3%). Approximately 19.6% ± 1.0% of the participants reported that they would cover the 5,000 THB emergency expense only with cash or cash equivalent without resorting to other means. Experience of economic distress was associated with anxiety disorder after adjusting for covariables (Adjusted Odds Ratio (OR) = 2.47; 95% CI [1.45–4.19]). There was no evidence that availability of emergency cash reserves significantly modified the stated association, nor the association between experience of economic distress and other outcomes. However, with regard to anxiety disorder, depressive symptoms and history of gambling in past 30 days, the p-for-trend values (p-for-trend < 0.001) suggested that those with emergency cash reserves had lower prevalence of these outcomes than those without emergency cash reserves.

**Conclusions:**

The study findings did not support our hypothesis that availability of emergency cash reserves modified the association between experience of economic distress and behavioral health outcomes. Nonetheless, the study findings can serve as potentially useful basic information for relevant stakeholders. Future studies should consider qualitative data collection and longitudinal study design in order to explore these associations at greater depths.

## Introduction

Behavioral health refers to the connection between behaviors and health and includes mental health, substance use, as well as health behaviors (Alvernia University, 2021). The COVID-19 pandemic has impacted not only physical health with more than 3.5 million deaths and 175 million illnesses (Worldometer, 2021), but has also impacted behavioral health of the global population.

The higher prevalence of anxiety and depressive symptoms during the COVID-19 pandemic compared to historical norms was found to be associated with employment loss and COVID-19 related concerns (Nelson et al., 2020). A survey during the early stage of the COVID-19 pandemic also found that anxiety and depression were associated with financial problems (Hertz-Palmor et al., 2021). These findings were similar to studies conducted prior to the pandemic, which found associations between financial problems and psychological distress (Richardson, Elliott & Roberts, 2013; Richardson et al., 2015; Khandelwal et al., 2018), alcohol and drug use (Richardson, Elliott & Roberts, 2013; Richardson et al., 2017), and tobacco use (Siahpush & Carlin, 2006).

The pandemic also induced the biggest global economic recession since the Great Depression (Zumbrun, 2020), resulting in business closures, employment losses, and worldwide economic distress. Studies have found associations between financial distress and depression (Ford et al., 2019), perceived stress, and worsened self-reported general health (Sweet et al., 2013). Based on the family stress model proposed by Pauline Boss (Boss, 2002), a given event or situation (including economic distress) can induce stress at varying degrees, depending on moderation by available resources and perception of the event or situation. A study based on the family stress model showed that economic pressure was associated with money-related stress (Prawitz, Kalkowski & Cohart, 2013), and the association was moderated by financial adjustments (*i.e*., use of available resources) and perception of the event as being within the internal locus within one’s ability to control, as opposed to an external locus outside of one’s control (*i.e*., perception).

Emergency cash reserves, also known as “precautionary savings” or “financial emergency fund”, refers to an amount of available cash to help meet “modest unexpected expenses—such as a car repair or a [leaking] roof”(Chen, 2019). Having emergency cash reserves is a type of resource for financial adjustment that has been shown to be associated with subjective well-being (Bell et al., 2014). Availability of emergency cash reserves may also enable individuals experiencing economic distress to perceive an unexpected expense as an issue within their internal locus of control (Prawitz & Cohart, 2016; Dwiastanti, 2017), which would lessen the level of stress and behavioral health issues that the distress can induce. Based on the reviewed literature, we hypothesize that availability of emergency cash reserves may help to moderate the association between economic distress and behavioral health outcomes.

Since 2013, bi-annual surveys commissioned by the US Federal Reserves has created a standardized instrument which measured the proportion of households with cash or cash equivalent reserves available to cover a small emergency of 400 US dollars (Board of Governors of the Federal Reserve System, 2019), a measure which had a potential to be replicated in other settings. The objective of this study was to assess the extent that the availability of emergency cash reserves modified the association between experience of economic distress during the COVID-19 pandemic and behavioral health outcomes. If the association between economic distress and behavioral health outcomes was indeed weaker among those with emergency cash reserves than among those without emergency cash reserves, then the findings of this study could provide preliminary basis for further investigation into the potential of maintaining emergency cash reserves as a measure to help create resiliency and mitigate the impact of economic distress on adverse behavioral health.

## Materials and Methods

### Study design and setting

We commissioned a survey research firm (Research Centre for Social and Business Development Co. Ltd. (SAB), Bangkok, Thailand) to conduct a phone-based cross-sectional study in late April 2021, during beginning of the third wave of the COVID-19 pandemic in Thailand, an upper-middle-income country in Southeast Asia.

### Study population and sample size calculation

The study population included Thai people aged 18 years and over in 15 provinces who had a cell phone number. Thailand currently has 94.8% mobile phone usage among population aged 6 years or older, with similar proportions across all socioeconomic groups (Thailand National Statistical Office, 2021). SAB investigators performed sample size calculation at 95% confidence interval, 3% margin of error, design effect of 1.2, and response rate of 80%, and obtained the final sample size of 1,537 respondents.

### Sampling methods

SAB conducted stratified two-stage sampling by dividing Thailand into five regions: Bangkok Metropolitan Area, the Central Region, the North, the Northeast, and the South. For each region, SAB researchers selected the study provinces using systematic sampling. SAB possessed a list of over 100,000 mobile phone numbers of users from all provinces of Thailand who were registered with the three major telecommunication operators (AIS, True-Move, DTAC), and sampled mobile phone number from the list of users in the selected provinces using cumulative systematic sampling.

### Measurement: availability of emergency cash reserves

We drafted the question to measure availability of emergency cash reserves based on the U.S. Federal Reserves’ Survey of Household Economic and Decision-Making 2018 (Board of Governors of the Federal Reserve System, 2019), which asked American participants how they would cover payment in an emergency which cost 400 US Dollars. The authors could not find such similar estimates or survey instruments prior to the current study. Given the exchange rate at the time of study (approximately 31 THB per 1 USD), we converted the amount to 12,400 THB. The amount was then adjusted for purchasing power using the ratio between Thailand’s GDP (PPP) *per capita* (World Bank, 2021b) of $19,277 and Thailand’s GDP (nominal) *per capita* (World Bank, 2021a) of $7,087, from which we obtained the ratio of 2.47. We calculated the emergency amount adjusted for purchasing power parity in the Thai context to be 12,400 THB/2.47 = 5,020 THB, rounded to 5,000 THB for ease of survey administration. We added the time frame (“within one week”) to standardize the sense of urgency, slightly modified the responses to suit the Thai context, and translated the question from English to Thai. We considered participants who answered that they would cover the emergency expense “*With the money in [their] savings account, cash, or other liquid assets* (*e.g*., *converting mutual funds to cash)*” without also answering that they would use their credit card, take out loans or lines of credits, or sell/pawn personal possessions to be those with available emergency cash reserves. Those who indicated that they refuse to answer the question were treated as having missing data.

### Measurement: experience of economic distress

We drafted economic distress measurement questions based on those used in a study on the association between financial stress and smoking (Siahpush & Carlin, 2006) and a study on financial stress and mental health conducted prior to the COVID-19 pandemic (Richardson et al., 2017) and during the COVID-19 pandemic (Hertz-Palmor et al., 2020). We modified the questions to suit the context of the COVID-19 pandemic in Thailand, created two groups of measurement (economic distress experienced since the declaration of the COVID-19 pandemic state of emergency, and economic distress experienced within the past 30 days prior to the survey) and translated the questions to Thai.

### Measurement: behavioral health outcomes

All behavioral health outcome measurement questions were available in validated instruments in Thai prior to designing our survey. We used Thai versions of the GAD-7 (Faculty of Medicine Ramathibodi Hospital, 2018) and the PHQ-2 (Department of Health, 2019) to assess prevalence of anxiety and depressive symptoms, respectively. The GAD-7 instrument in our study had relatively high reliability (Cronbach’s alpha = 0.85; 95% CI [0.83–0.87]). The validity of the Thai and original versions of these instruments had been validated in previous studies (Löwe et al., 2008, p. 7; Lotrakul, Sumrithe & Saipanish, 2008). We used the cut-off points of ≥10 points (range: 0–21 points) for the GAD-7 and at ≥3 points (range: 0–6 points) for the PHQ-2. These standardized screening tools were designed to measure anxiety and depressive symptoms at the time of the study based only on self-reported personal experience during the 2-weeks period prior to the survey.

We modified questions on weekly exercise, sleeping behaviors, gambling, and tobacco use from the self-reported National School Survey on Alcohol Consumption, Substance Use and Other Health-Risk Behaviors (Paileeklee et al., 2016). We modified questions on alcohol consumption, including binge-drinking (having five or more standard drinks in one drinking session), from the Thailand Smoking and Drinking Behaviour Survey 2017 face-to-face interview questionnaire (National Statistical Office, 2018). The survey research firm further modified the wording of the questions to suit the phone-based survey during and after pilot-testing the instrument.

### Data collection and quality control

Phone-based data collectors of the research firm were the research company’s regular researchers who received training on the use of the study instrument and data collection methods. The survey research firm employed a total of 10 data collection teams of four to five members and one supervisor each. Each team supervisor was responsible for assessing the validation and completeness of each questionnaire, and reported the progress of data collection at the end of each workday. Each data collection team member was required to work only for the region assigned to the team. In order to control for the effect of variation in assigned regions, we instructed data collectors to administer the survey only in the official (central) dialect of the Thai language and to administer the interview questions strictly as they appeared on the questionnaire. During each telephone call, the data collection team member filled the call log in order to record the response rate. Each number successfully dialed but without response would be repeated at least three times. Prior to administration of the survey, each data collector would ask for verbal informed consent from the potential study participant using a standardized script. In case of non-response or refusal, the data collector would try other numbers based on the survey research firm’s replacement sampling models.

### Data collection supervision and data cleaning

The survey research firm assigned a minimum target number of responses from respondents in each province to each data collection team, and assigned team supervisors to monitor and report on their team’s progress daily. Data collection teams used a software package to detect and prevent data entry errors and data inconsistencies. Team supervisors also performed initial validation during data collection and data entry. After data collection, the survey research firm excluded questionnaires with missing information on the respondent’s sex, age, province, and the questions pertaining to the primary objective of the survey.

### Statistical analyses

To describe the characteristics of the study population, we used descriptive statistics with adjustment for regional-level sampling weights and complex survey design. We calculated the sampling weight of each participant based on the ratio between the population in each region and the number of samples from the region. We specified region as the stratification variable and specified the unique ID variable when we calculated weighted analyses in order to account for complex survey design. We performed logistic multivariate regression analyses with adjustment for demographic and socioeconomic characteristics in a similar manner as in previous studies (Siahpush & Carlin, 2006; Hertz-Palmor et al., 2020). We also assessed additive and multiplicative effect modification in each association using relative excess risk due to interaction (RERI) and multiplicative interaction terms, as well as p-for-trend, pseudo-R-squared, and the likelihood ratio (-2LL) values for Adjusted OR. We considered additive interaction to be present at 95% level of confidence if the 95% confidence interval of the RERI did not include the null value of 0. We considered multiplicative interaction to be present at 95% level of confidence if the 95% confidence interval for the multiplication interaction term did not include the null value of 1. If the p-for-trend was less than 0.05, we considered that there was a linear trend by the presented categories. The pseudo-R-squared and the likelihood ratio (-2LL) values were included as a measure of goodness-of-fit in order to compare an adjusted OR model for a given outcome to the others. Pseudo R-squared values can be interpreted as indicators of how well each model fit and was able to explain variations in the outcome. Generally, the higher the pseudo R-squared value is, the better the model will be. However, the pseudo R-squared may also be artificially inflated if independent variables are highly correlated with one another (*i.e*., there is collinearity). Likelihood ratio (-2LL) value refers to the extent that the variables in the model help explain the distribution of the outcome better than the outcome itself. For negative likelihood ratios, the further the ratio is less than −1, the better the model tends to be at explaining the outcome. All analyses were made with R version 4.0.3.

Furthermore, as our definition of having available emergency cash reserves differed from the criteria used in the original study by the U.S. Federal Reserves, we decided to perform a sensitivity analysis to assess the extent that such differences influenced the study findings. In this sensitivity analysis, we considered those who answered that they would cover the 5,000 THB emergency expense “With the money in [their] savings account, cash, or other liquid assets (*e.g*., converting mutual funds to cash)” or “Put it on [their] credit card and pay it off in full at the next statement” without also answering other options as those with available emergency cash reserves. We presented the sensitivity analysis findings in a separate results table.

### Ethical considerations

The contracted survey research firm routinely conducted public opinion polls on behalf of various government agencies and academic institutions. Specifically for this survey, investigators also prepared an additional script for verbal informed consent for potential study participants during initial contact, in which the phone-based data collectors informed the potential participants of the name of the research firm and investigator’s organization, the title of the study, the approximate length of the survey (5 min), assessed whether the potential participants were 18 years of age or older, informed the potential participants of their rights to refuse to answer any question or stop the interview at any time, that there was no compensation for the study participants, and provided the names and contact number of the project manager and the project coordinator. The data collector then asked the potential participant whether they had question, prior to obtaining verbal informed consent and asked for permission to conduct follow-up data collection, should there be a follow-up survey.

The research firm did not collect the participant’s name or identifiable information in the survey or include such information in the data set. The verbal informed consent processes and data collection procedures were designed in accordance with the standards outlined in the Helskinki Declaration of 1975 (2013 revision). The investigators have received approval for analysis of anonymized secondary data from this survey (Faculty of Medicine Human Research Ethic Committee, Prince of Songkla University; REC. 62-054-18-1).

## Results

Among the potential participants, the response rate was 68.3% (16.2% of potential participants refused to participate, 15.5% could not be reached after three attempts). A total of 1,555 individuals from 15 of 76 provinces of Thailand completed our study interview.

### Characteristics of the study participants

Univariate descriptive analyses showed that most participants were female, with mean age of 41 years, and married with child(ren) (Table 1). Two-thirds of the participants finished high school or less, and about one-fourth finished bachelor’s degree or higher. More than three-fourths of the participants earned 20,000 THB per month or less in personal income. In order to cover 5,000 THB in emergency expense, less than one-fifth of the participants indicated that they would only use money in their savings account, cash or liquid assets to cover the expenses. More than half of the participants indicated that they had experienced at least one economic distress since the declaration of the COVID-19 pandemic state of emergency and within the past 30 days, with loss of work or primary source of income being the most common type of distress. The proportions of those who screened positive for depressive symptoms and those who screened positive for generalized anxiety disorder were 8.5% and 5.8%, respectively. One-sixth of the respondents reported that they smoked within the past 30 days, and 3% reported binge-drinking within the past 30 days.

**Table 1:** General characteristics of the study participants (*n* = 1,555 persons).

Characteristic	Weighted percent ± Standard errors
**Gender**	
Male	48.3% **±** 1.3%
Female	51.7% **±** 1.3%
Age (years) (mean **±** SD)	41.3 **±** 0.3 years
**Marital Status**	
Single	30.5% ± 1.2%
Married with child(ren)	59.8% ± 1.2%
Married, no children	4.5% ± 0.5%
Widowed/divorced/separated	5.3% ± 0.6%
**Highest Level of Education Completed**	
Junior High School or Lower	37.0% ± 1.2%
High School or Equivalent	27.7%± 1.1%
Associate’s Degree or Equivalent	9.8% ± 0.8%
Bachelor’s Degree	23.9% ± 1.1%
Graduate Degree	1.6% ± 0.3%
**Personal Monthly Income**	
No more than 5,000 THB	9.6% ± 0.8%
5,001–10,000 THB	25.6% ± 1.1%
10,001–20,000 THB	42.0% ± 1.3%
20,001–30,000 THB	15.3% ± 0.9%
30,001–40,000 THB	5.2% ± 0.6%
40,001–50,000 THB	1.4% ± 0.3%
More than 50,000 THB	0.8% ± 0.2%
**Emergency Expense:** Suppose that you have an emergency expense that costs 5,000 THB, how would you pay for this expense within one week? (*multiple responses allowed*)	
Put it on my credit card and pay it off in full at the next statement	1.0% ± 0.3%
Put it on my credit card and pay it off in installments	2.0% ± 0.4%
With the money in my savings account, cash, or other liquid assets	34.0% ± 1.1%
Using money from a bank loan or line of credit	3.3% ± 1.1%
Borrowing from friends or family members	51.6% ± 1.2%
Using a payday loan, deposit advance, or overdraft	9.0% ± 0.7%
Selling a personal belonging	17.4% ± 0.9%
Pawning a personal belonging	21.4% ± 1.0%
I would not be able to find money to pay for the expense	7.3% ± 0.7%
Participant with emergency cash reserves available*	19.6% ± 1.0%
**Experience of Economic Distress in the Past 30 Days**	
Lost work or primary source of income for three months or longer	38.6% ± 1.2%
Had reduced pay or days of work	17.0% ± 0.9%
Been unable to pay for electricity, water, or telephone bills on time	20.8% ± 1.0%
Been unable to make mortgage payment/car payment/installments/rent payments on time	27.8% ± 1.1%
Pawned or sold something in order to raise enough cash to pay for necessity	19.8% ± 1.0%
Gone without meals at least once	1.7% ± 0.3%
Asked for financial help from friends or family	22.8% ± 1.1%
Asked for help from welfare/community organization	1.0% ± 0.3%
Experienced at least 1 economic distress in past 30 days	58.9% ± 1.3%
**Mental Health Outcomes**	
Screened positive for general anxiety disorder**	5.8% ± 0.6%
Screened positive for depressive symptoms**	8.5% ± 0.7%
Exercised at least once per week within the past 30 days	48.3% ± 1.3%
Slept at least 6 hours per night within the past 30 days	92.0% ± 0.7%
Gambled in past 30 days (including lottery)	54.8% ± 1.2%
Smoked within past 30 days	14.5% ± 0.9%
Binged-drank within the past 30 days	3.4% ± 0.5%

**Notes:**

* Participant answered only “*With the money in my savings account, cash, or other liquid assets (*e.g*., converting mutual funds to cash)*” with no other choice.

** Anxiety: GAD-7 >= 10 points; Depressive symptoms: PHQ-2 >= 3 points.

### Association between experience of economic distress and behavioral health outcomes

Cross-tabulation of experience of economic distress and behavioral health outcomes showed that anxiety disorder, depressive symptoms, and history of gambling in past 30 days were higher among participants who experienced economic distress within past 30 days than those who did not (Table 2). However, the associations with depressive symptoms and history of gambling were not statistically significant after adjusting for participants’ sex, age, marital status, education level, and personal monthly income. Only anxiety disorder remained significantly associated with experience of economic distress after adjusting for covariables (Adjusted OR = 2.47; 95% CI [1.45–4.19]).

**Table 2:** Prevalence of behavioral health outcomes according to experience of economic distress within past 30 days (*n* = 1,498 participants).

Outcome	Weighted prevalence (Percent ± SE)	Unadjusted OR (95% CI)	Adjusted OR* (95% CI)	Pseudo-R-squared, and the likelihood ratio (-2LL) for Adjusted OR
***Anxiety disorder* (GAD-7 ≥ 10 points)**				
Did not experience distress (*n* = 596)	3.2% ± 0.7%	1.0 (Ref.)	1.0 (Ref.)	Pseudo R^2^ = 0.056
Experienced distress (*n* = 902)	7.6% ± 0.9%	**2.54 [1.53–4.22]**	**2.47 [1.45–4.19]**	-2LL = −320.32
***Depressive symptoms* (PHQ-2 ≥ 3 points)**				
Did not experience distress (*n* = 596)	6.7% ± 1.0%	1.0 (Ref.)	1.0 (Ref.)	Pseudo R^2^ = 0.029
Experienced distress (*n* = 902)	9.7% ± 1.0%	**1.51 [1.03–2.21]**	1.44 [0.96–2.17]	-2LL = −429.33
***Exercising at least once per week in past 30 days***				
Did not experience distress (*n* = 596)	48.9% ± 2.0%	1.0 (Ref.)	1.0 (Ref.)	Pseudo R^2^ = 0.027
Experienced distress (*n* = 902)	48.1% ± 1.6%	0.97 [0.79–1.19]	1.12 [0.91–1.39]	-2LL = −1,026.50
***Slept at least 6 hours per night in past 30 days***				
Did not experience distress (*n* = 596)	93.5% ± 1.0%	1.0 (Ref.)	1.0 (Ref.)	Pseudo R^2^ = 0.044
Experienced distress (*n* = 902)	90.9% ± 1.0%	0.70 [0.47–1.03]	0.77 [0.52–1.15]	-2LL = −402.69**
***Gambled in past 30 days (including lottery)***				
Did not experience distress (*n* = 596)	50.1% ± 2.0%	1.0 (Ref.)	1.0 (Ref.)	Pseudo R^2^ = 0.037
Experienced distress (*n* = 902)	58.1% ± 1.6%	**1.38 [1.12–1.70]**	1.23 [0.99–1.53]	-2LL = −994.08**
***Smoked in past 30 days***				
Did not experience distress (*n* = 596)	12.7% ± 1.3%	1.0 (Ref.)	1.0 (Ref.)	Pseudo R^2^ = 0.037
Experienced distress (*n* = 902)	16.0% ± 1.2%	1.31 [0.98–1.76]	1.20 [0.85–1.67]	-2LL = −482.31
***Binge-drank in past 30 days***				
Did not experience distress (*n* = 596)	4.4% ± 0.8%	1.0 (Ref.)	1.0 (Ref.)	Pseudo R^2^ = 0.129
Experienced distress (*n* = 902)	2.8% ± 0.5%	0.61 [0.35–1.06]	0.67 [0.37–1.22]	-2LL = −197.47

**Notes:**

* Adjusted OR (95% CI) included adjustments for sex, age, marital status, education level, and personal monthly income.

** Weighted likelihood ratio (-2LL) could not be displayed because “models could not be fitted to the same size of dataset”; unweighted -2LL displayed instead.

Bold numbers denote statistical significance at 95% level of confidence.

### Modification in the association between experience of economic distress and behavioral health outcome by availability of emergency cash reserves

Assessment of variations in the association between experience of economic distress in past 30 days and behavioral health outcomes also showed similar results (Table 3). There was no statistically significant indication of additive or multiplicative effect modifications (*i.e*., moderation). Additive and multiplicative interaction terms were all non-significant at 95% level of confidence. There was also no statistically significant heterogeneity in the association between experience of economic distress and behavioral health outcomes when the analyses were stratified by availability of emergency cash reserves (all Breslow-Day Test of heterogeneity *p*-values > 0.05). When we conducted further exploratory analyses, we found significant descending trends (p-for-trend < 0.001) in prevalence of anxiety, depressive symptoms, and history of gambling in past 30 days in the following order: (1) those without emergency cash reserves who experienced economic distress; (2) those without emergency cash reserves who did not experience economic distress; (3) those with emergency cash reserves who experienced economic distress; (4) those with emergency cash reserves who did not experience economic distress. Notably, participants who experienced economic distress but had emergency cash reserves had lower prevalence of the outcomes than participants who did not experience economic distress but had no emergency cash reserves (3.7% *vs*. 4.2% for anxiety, 3.7% *vs*. 7.8% for depressive symptoms, and 41.5% *vs*. 55.2% for gambling (including lottery) in past 30 days). In sensitivity analysis using an alternative set of criteria for having emergency cash reserves (Table 4), the findings were largely similar to those in Table 3. Indications of additive and multiplicative effect modifications were all statistically non-significant at 95% level of confidence, and there was no statistically significant heterogeneity in the association between economic distress and behavioral health outcome when stratified by availability of emergency cash reserves (all Breslow-Day-Test *p*-values > 0.05).

**Table 3:** Prevalence of behavioral health outcomes by according to experience of economic distress within past 30 days, stratified by availability of emergency cash reserves.

Outcome	Weighted prevalence (Percent ± SE)	Unadjusted OR (95% CI)	Adjusted OR* (95% CI)	P-for-trend, pseudo-R-squared, the likelihood ratio (-2LL) for Adjusted OR, and the *p*-value of the Breslow-Day test on Homogeneity of Odds Ratios
***Anxiety disorder* (GAD-7 ≥ 10 points *)***				
*Participants with emergency cash reserves (n = 295)*				
Had emergency cash reserves, did not experience distress (*n* = 187)	1.6% ± 0.9%	1.0 (Ref.)	1.0 (Ref.)	**P-for trend <0.001**
Had emergency cash reserves, experienced distress (*n* = 108)	3.7% ± 1.8%	2.39 [0.53–10.87]	3.20 [0.55–18.79]	Pseudo R^2^ = 0.163 -2LL = −27.67**
*Participants with no emergency cash reserves (n = 1,203)*				
No emergency cash reserves, did not experience distress (*n* = 409)	4.2% ± 1.0%	1.0 (Ref.)	1.0 (Ref.)	Pseudo R^2^ = 0.050 -2LL = −281.30**
No emergency cash reserves, experienced distress (*n* = 794)	8.1% ± 1.0%	**2.03 [1.17–3.52]**	**1.93 [1.09–3.41]**	Breslow-Day Test *p*-value = 0.851
***Depressive symptoms (*PHQ-2 ≥ 3 points*)***				
*Participants with emergency cash reserves (n = 295)*				
Had emergency cash reserves, did not experience distress (*n* = 187)	2.2% ± 1.1%	1.0 (Ref.)	1.0 (Ref.)	**P-for trend <0.001**
Had emergency cash reserves, experienced distress (*n* = 108)	3.7% ± 1.8%	1.75 [0.43–7.16]	2.33 [0.86–6.31]	Pseudo R^2^ = 0.138 -2LL = −31.58**
*Participants with no emergency cash reserves (n = 1,203)*				
No emergency cash reserves, did not experience distress (*n* = 409)	7.8% ± 1.3%	1.0 (Ref.)	1.0 (Ref.)	Pseudo R^2^ = 0.031 -2LL = −362.26**
No emergency cash reserves, experienced distress (*n* = 794)	10.4% ± 1.1%	1.36 [0.89–2.08]	1.37 [0.87–2.14]	Breslow-Day Test *p*-value = 0.728
***Exercising at least once per week in past 30 days***				
*Participants with emergency cash reserves (n = 295)*				
Had emergency cash reserves, did not experience distress (*n* = 187)	58.1% ± 3.6%	1.0 (Ref.)	1.0 (Ref.)	P-for trend = 0.347
Had emergency cash reserves, experienced distress (*n* = 108)	51.8% ± 4.8%	0.78 [0.48–1.25]	0.91 [0.54–1.54]	Pseudo R^2^ = 0.044 -2LL = −191.01**
*Participants with no emergency cash reserves (n = 1,203)*				
No emergency cash reserves, did not experience distress (*n* = 409)	46.1% ± 2.5%	1.0 (Ref.)	1.0 (Ref.)	Pseudo R^2^ = 0.029 -2LL = −798.60**
No emergency cash reserves, experienced distress (*n* = 794)	47.4% ± 1.8%	1.06 [0.83–1.34]	1.19 [0.93–1.53]	Breslow-Day Test *p*-value = 0.261
***Slept at least 6 hours per night in past 30 days***				
*Participants with emergency cash reserves (n = 295)*				
Had emergency cash reserves, did not experience distress (*n* = 187)	91.4% ± 2.1%	1.0 (Ref.)	1.0 (Ref.)	P-for trend = 0.855
Had emergency cash reserves, experienced distress (*n* = 108)	90.4% ± 2.9%	0.89 [0.39–2.04]	1.12 [0.47–2.68]	Pseudo R^2^ = 0.067 -2LL = −81.48**
*Participants with no emergency cash reserves (n = 1,203)*				
No emergency cash reserves, did not experience distress (*n* = 409)	94.3% ± 1.2%	1.0 (Ref.)	1.0 (Ref.)	Pseudo R^2^ = 0.068 -2LL = −303.19**
No emergency cash reserves, experienced distress (*n* = 794)	90.9% ± 1.0%	**0.60 [0.37–0.98]**	0.62 [0.38–1.02]	Breslow-Day Test *p*-value = 0.422
***Gambled in past 30 days (including lottery)***				
*Participants with emergency cash reserves (n = 295)*				
Had emergency cash reserves, did not experience distress (*n* = 187)	36.7% ± 3.6%	1.0 (Ref.)	1.0 (Ref.)	**P-for trend <0.001**
Had emergency cash reserves, experienced distress (*n* = 108)	41.5% ± 4.8%	1.22 [0.75–2.00]	1.01 [0.58–1.76]	Pseudo R^2^ = 0.045 -2LL = −180.70**
*Participants with no emergency cash reserves (n = 1,203)*				
No emergency cash reserves, did not experience distress (*n* = 409)	55.2% ± 2.5%	1.0 (Ref.)	1.0 (Ref.)	Pseudo R^2^ = 0.041 -2LL = −763.09**
No emergency cash reserves, experienced distress (*n* = 794)	60.2% ± 1.7%	1.23 [0.96–1.57]	1.13 [0.88–1.45]	Breslow-Day Test p-value = 0.985
***Smoked in past 30 days***				
*Participants with emergency cash reserves (n=295)*				
Had emergency cash reserves, did not experience distress (*n* = 187)	10.7% ± 2.3%	1.0 (Ref.)	1.0 (Ref.)	P-for trend = 0.206
Had emergency cash reserves, experienced distress (*n* = 108)	11.1% ± 3.0%	1.05 [0.49–2.24]	1.00 [0.42–2.42]	Pseudo R^2^ = 0.307 -2LL = N/A***
*Participants with no emergency cash reserves (n =1,203)*				
No emergency cash reserves, did not experience distress (*n* = 409)	14.0% ± 1.7%	1.0 (Ref.)	1.0 (Ref.)	Pseudo R^2^ = 0.248 -2LL = −387.21**
No emergency cash reserves, experienced distress (*n* = 794)	16.4% ± 1.3%	1.21 [0.86–1.69]	1.11 [0.75–1.65]	Breslow-Day Test *p*-value = 0.730
***Binge-drank in past 30 days***				
*Participants with emergency cash reserves (n = 295)*				
Had emergency cash reserves, did not experience distress (*n* = 187)	4.9% ± 1.6%	1.0 (Ref.)	1.0 (Ref.)	P-for trend = 0.086
Had emergency cash reserves, experienced distress (*n* = 108)	3.7% ± 1.8%	0.76 [0.23–2.52]	0.60 [0.15–2.41]	Pseudo R^2^ = 0.351 -2LL = N/A***
*Participants with no emergency cash reserves (n=1,203)*				
No emergency cash reserves, did not experience distress (n=409)	4.4% ± 1.0%	1.0 (Ref.)	1.0 (Ref.)	Pseudo R^2^ = 0.100 -2LL = −151.46
No emergency cash reserves, experienced distress (n=794)	2.7% ± 0.6%	0.59 [0.31–1.12]	0.66 [0.32–1.37]	Breslow-Day Test *p*-value = 0.718

**Notes:**

* Adjusted OR (95% CI) included adjustments for sex, age, marital status, education level, and personal monthly income.

** Weighted likelihood ratio (-2LL) could not be displayed because “models could not be fitted to the same size of dataset”; unweighted -2LL displayed instead.

*** Statistical program would not perform the test; “Likelihood gets worse with more variables.”

Bold numbers denote statistical significance at 95% level of confidence.

**Anxiety:** Additive interaction term RERI (95% CI) = −0.46 [−1.73 to 0.81]; Multiplicative interaction term Adj OR (95% CI) = 1.23 [0.24–6.20].

**Depressive symptoms:** Additive interaction term RERI (95% CI) = −0.18 [−0.96 to 0.60]; Multiplicative interaction term Adj OR (95% CI) = 1.19 [0.28–5.15].

**Exercising:** Additive interaction term RERI (95% CI) = −0.23 [−0.97 to 0.52]; Multiplicative interaction term Adj OR (95% CI) = 0.82 [0.47–1.42].

**Sleeping:** Additive interaction term RERI (95% CI) = 0.37 [−0.20 to 0.94]; Multiplicative interaction term Adj OR (95% CI) = 1.57 [0.59–4.21].

**Gambling**: Additive interaction term RERI (95% CI) = −0.09 [−0.46 to 0.28]; Multiplicative interaction term Adj OR (95% CI) = 0.95 [0.53–1.70].

**Smoking:** Additive interaction term RERI (95% CI) = −0.23 [−1.05 to 0.60]; Multiplicative interaction term Adj OR (95% CI) = 0.80 [0.32–2.01].

**Binge-Drinking:** Additive interaction term RERI (95% CI) = −0.04 [−1.57 to 1.49]; Multiplicative interaction term Adj OR (95% CI) = 1.07 [0.25–4.51].

**Table 4:** Prevalence of behavioral health outcomes according to experience of economic distress within past 30 days, stratified by availability of emergency cash reserves (alternative definition).

Outcome	Weighted prevalence (Percent ± SE)	Unadjusted OR (95% CI)	Adjusted OR* (95% CI)	P-for-trend, pseudo-R-squared, and the likelihood ratio (-2LL) for Adjusted OR
***Anxiety disorder (*GAD-7 ≥ 10 points*)***				
*Participants with emergency cash reserves (n = 304)*				
Had emergency cash reserves, did not experience distress (*n* = 194)	1.5% ± 0.9%	1.0 (Ref.)	1.0 (Ref.)	**P-for trend <0.001**
Had emergency cash reserves, experienced distress (*n* = 110)	3.7% ± 1.8%	2.43 [0.54–11.07]	3.22 [0.55–18.68]	Pseudo R^2^ = 0.162 -2LL = −27.88**
*Participants with no emergency cash reserves (n = 1,194)*				
No emergency cash reserves, did not experience distress (*n* = 402)	4.2% ± 1.0%	1.0 (Ref.)	1.0 (Ref.)	Pseudo R^2^ = 0.049 -2LL = −280.79**
No emergency cash reserves, experienced distress (*n* = 792)	8.1% ± 1.0%	**2.00 [1.16–3.46]**	**1.90 [1.08–3.37]**	Breslow-Day Test *p*-value = 0.819
***Depressive symptoms (*PHQ-2 ≥ 3 points *)***				
*Participants with emergency cash reserves (n = 304)*				
Had emergency cash reserves, did not experience distress (*n* = 194)	2.6% ± 1.1%	1.0 (Ref.)	1.0 (Ref.)	**P-for trend <0.001**
Had emergency cash reserves, experienced distress (*n* = 110)	3.7% ± 1.8%	1.42 [0.37–5.41]	1.35 [0.41–4.45]	Pseudo R^2^ = 0.099 -2LL = −36.44**
*Participants with no emergency cash reserves (n = 1,194)*				
No emergency cash reserves, did not experience distress (*n* = 402)	7.7% ± 1.3%	1.0 (Ref.)	1.0 (Ref.)	Pseudo R^2^ = 0.031 -2LL = −359.01**
No emergency cash reserves, experienced distress (*n* = 792)	10.4% ± 1.1%	1.39 [0.90–2.13]	1.41 [0.90–2.21]	Breslow-Day Test *p*-value = 0.965
***Exercising at least once per week in past 30 days***				
*Participants with emergency cash reserves (n = 304)*				
Had emergency cash reserves, did not experience distress (*n* = 194)	58.6% ± 3.5%	1.0 (Ref.)	1.0 (Ref.)	P-for trend = 0.311
Had emergency cash reserves, experienced distress (*n* = 110)	51.8% ± 4.8%	0.76 [0.47–1.22]	0.90 [0.54–1.52]	Pseudo R^2^ = 0.04 -2LL = −197.62**
*Participants with no emergency cash reserves (n=1,194)*				
No emergency cash reserves, did not experience distress (*n* = 402)	45.6% ± 2.5%	1.0 (Ref.)	1.0 (Ref.)	Pseudo R^2^ = 0.027 -2LL = −793.28**
No emergency cash reserves, experienced distress (*n* = 792)	47.4% ± 1.8%	1.07 [0.84–1.37]	1.21 [0.94–1.55]	Breslow-Day Test *p*-value = 0.201
***Slept at least 6 hours per night in past 30 days***				
*Participants with emergency cash reserves (n = 304)*				
Had emergency cash reserves, did not experience distress (*n* = 194)	91.7% ± 2.0%	1.0 (Ref.)	1.0 (Ref.)	P-for trend = 0.947
Had emergency cash reserves, experienced distress (*n* = 110)	90.6% ± 2.8%	0.87 [0.38–1.99]	1.08 [0.45–2.59]	Pseudo R^2^ = 0.064 -2LL = −82.49
*Participants with no emergency cash reserves (n=1,194)*				
No emergency cash reserves, did not experience distress (*n* = 402)	94.2% ± 1.2%	1.0 (Ref.)	1.0 (Ref.)	Pseudo R^2^ = 0.067 -2LL = −302.73
No emergency cash reserves, experienced distress (*n* = 792)	90.9% ± 1.0%	**0.61 [0.38–1.00]**	0.63 [0.38–1.04]	Breslow-Day Test *p*-value = 0.464
***Gambled in past 30 days (including lottery)***				
*Participants with emergency cash reserves (n = 304)*				
Had emergency cash reserves, did not experience distress (*n* = 194)	36.9% ± 3.5%	1.0 (Ref.)	1.0 (Ref.)	**P-for trend <0.001**
Had emergency cash reserves, experienced distress (*n* = 110)	42.6% ± 4.8%	1.27 [0.78–2.06]	1.05 [0.61–1.81]	Pseudo R^2^ = 0.042 -2LL = −187.66**
*Participants with no emergency cash reserves (n = 1,194)*				
No emergency cash reserves, did not experience distress (*n* = 402)	55.4% ± 2.5%	1.0 (Ref.)	1.0 (Ref.)	Pseudo R^2^ = 0.041 -2LL = −757.68**
No emergency cash reserves, experienced distress (*n* = 792)	60.1% ± 1.7%	1.21 [0.95–1.55]	1.12 [0.87–1.44]	Breslow-Day Test *p*-value = 0.876
***Smoked in past 30 days***				
*Participants with emergency cash reserves (n = 304)*				
Had emergency cash reserves, did not experience distress (*n* = 194)	11.3% ± 2.3%	1.0 (Ref.)	1.0 (Ref.)	P-for trend = 0.270
Had emergency cash reserves, experienced distress (*n* = 110)	10.9% ± 3.0%	0.96 [0.46–2.03]	0.97 [0.40–2.34]	Pseudo R^2^ = 0.317 -2LL = −72.42**
*Participants with no emergency cash reserves (n = 1,194)*				
No emergency cash reserves, did not experience distress (*n* = 402)	13.8% ± 1.7%	1.0 (Ref.)	1.0 (Ref.)	Pseudo R^2^ = 0.249 -2LL = −382.97**
No emergency cash reserves, experienced distress (*n* = 792)	16.5% ± 1.3%	1.24 [0.88–1.74]	1.14 [0.77–1.71]	Breslow-Day Test *p*-value = 0.539
***Binge-drank in past 30 days***				
*Participants with emergency cash reserves (n = 304)*				
Had emergency cash reserves, did not experience distress (*n* = 194)	4.7% ± 1.5%	1.0 (Ref.)	1.0 (Ref.)	P-for trend = 0.103
Had emergency cash reserves, experienced distress (*n* = 110)	3.7% ± 1.8%	0.77 [0.23–2.57]	0.60 [0.15–2.45]	Pseudo R^2^ = 0.352 -2LL = N/A***
*Participants with no emergency cash reserves (n = 1,194)*				
No emergency cash reserves, did not experience distress (*n* = 402)	4.5% ± 1.0%	1.0 (Ref.)	1.0 (Ref.)	Pseudo R^2^ = 0.100 -2LL = −151.15**
No emergency cash reserves, experienced distress (*n* = 792)	2.7% ± 0.6%	0.58 [0.31–1.10]	0.66 [0.32–1.36]	Breslow-Day Test *p*-value = 0.680

**Notes:**

* Adjusted OR (95% CI) included adjustments for sex, age, marital status, education level, and personal monthly income

** Weighted likelihood ratio (-2LL) could not be displayed because “models could not be fitted to the same size of dataset”; unweighted -2LL displayed instead

*** Statistical program would not perform the test; “Likelihood gets worse with more variables.”

Bold numbers denote statistical significance at 95% level of confidence.

**Anxiety:** Additive interaction term RERI (95% CI) = −0.45 [−1.68 to 0.79]; Multiplicative interaction term Adj OR (95% CI) = 1.27 [0.25–6.37].

**Depressive symptoms:** Additive interaction term RERI (95% CI) = −0.28 [−1.10 to 0.54]; Multiplicative interaction term Adj OR (95% CI) = 0.94 [0.23–3.81].

**Exercising:** Additive interaction term RERI (95% CI) = −0.27 [−1.02 to 0.48]; Multiplicative interaction term Adj OR (95% CI) = 0.79 [0.46–1.37].

**Sleeping:** Additive interaction term RERI (95% CI) = 0.34 [−0.25 to 0.94]; Multiplicative interaction term Adj OR (95% CI) = 1.50 [0.56–4.05].

**Gambling**: Additive interaction term RERI (95% CI) = −0.06 [−0.43 to 0.31]; Multiplicative interaction term Adj OR (95% CI) = 1.00 [0.57–1.78].

**Smoking:** Additive interaction term RERI (95% CI) = −0.34 [−1.21 to 0.53]; Multiplicative interaction term Adj OR (95% CI) = 0.71 [0.29–1.77].

**Binge-Drinking:** Additive interaction term RERI (95% CI) = −0.01 [−1.48 to 1.47]; Multiplicative interaction term Adj OR (95% CI) = 1.10 [0.26–4.62].

## Discussion

In this cross-sectional study, we described the extent that availability of emergency cash reserves modified the associations between experience of economic distress during the COVID-19 pandemic and behavioral health outcomes. We did not find statistically significant effect modifications, although there were linear trends in the prevalence of anxiety, depressive symptoms, and history of gambling in stratified analyses. Furthermore, considering the call for collaborative works between behavioral health and complementary fields (Ford et al., 2019), the findings of this study may have implications for stakeholders in behavioral health and economics.

We estimated that less than 20% of the Thai population would be able to cover a 5,000 THB ($160 US) emergency expense with cash or other liquid assets without needing to resort to loans, borrowing, or selling personal items. This was lower than in the United States, where 40% of the population had emergency cash reserves of similar purchasing power (Board of Governors of the Federal Reserve System, 2019). Unlike the US Federal Reserves’ study, our definition of having the required amount of emergency cash reserves did not include the use of credit card with repayment at the end of the month, as the use of bank-based credit cards in Thailand was relatively uncommon and confined primarily to the middle class (Krungsri Ayutthaya Bank, 2021). Although our sensitivity analyses showed virtually no difference in the study findings, future studies should consider revising the definition of having emergency cash reserves available if there are changes in the context of credit card use in Thailand.

We did not find evidence of moderation (*i.e*., effect modification) in the association between experience of economic distress and behavioral health outcomes by availability of emergency cash reserves to support our hypothesis based on the family stress model (Boss, 2002). However, comparison of the overall adjusted OR to the stratified adjusted OR for anxiety disorder and depressive symptoms showed that the overall adjusted OR was between the two strata, which was what we would expect from effect modifiers. Thus the absence of statistical evidence for the moderation should not be considered as evidence that the family stress model was not relevant for the study population.

In our analyses, the p-for-trend values indicated that participants with emergency cash reserves had lower prevalence of anxiety disorder and depressive symptoms than participants who did not have emergency cash reserves. The availability of emergency cash reserves (even in relatively small amounts) could have increased the confidence in ability to absorb the shock from economic distress (Gjertson, 2016) and contributed to the lower prevalence of anxiety disorder and depressive symptoms. Future studies should consider using in-depth interviews to gain further insights on the nature and mechanisms of such variations.

With regard to the outcomes, we presented prevalence of anxiety disorder and depressive symptoms as binary outcome using the same cut-off points for GAD-7 and PHQ-2 as in previous studies (Nelson et al., 2020; Hertz-Palmor et al., 2021) in order to allow for higher statistical power. However, anxiety disorder and depressive symptoms occur on a gradient of intensity, and future studies should consider analyzing the scores of the screening tests as continuous variables when assessing the association between economic distress and anxiety disorder and depressive symptoms.

Our cross-sectional design did not allow us to rule out reverse causality. Thus it was possible that participants who had depression became less likely to work, did not earn adequate income, and effectively did not have emergency savings. However, if experiences of economic distress were indeed stressors for anxiety and depression, then the prevalent anxiety disorder and depressive symptoms also could have been presentations of adjustment disorder with anxiety or depressed mood (Zelviene & Kazlauskas, 2018). Future studies should consider adapting a longitudinal study design in order to reduce potential reverse causation, and also consider inclusion of measurement of adjustment disorder and a global measure of stress.

The question on gambling in our study included the lottery, which is the most common form of gambling in Thailand (Assanangkornchai et al., 2016). Considering that other forms of gambling are illegal in the kingdom, the study findings should be interpreted in this context rather than the context of problem gambling, which is more commonly reported in the literatures (Johansson et al., 2009). The p-for-trend value also indicated that participants with emergency cash reserves had significantly lower history of gambling than participants without emergency cash reserves. This difference in prevalence of past-month gambling might have reflected the differences in the psychology of personal finance between individuals (Housel, 2020), and this issue should be explored at greater depths. Furthermore, it was not possible for us to determine whether the gambling behavior occurred after experiencing economic distress as a negative coping mechanism, or gambling itself was a factor that contributed to economic distress. Future studies should consider adopting the longitudinal study design, separating lottery purchase from other forms of gambling, and assessing the prevalence of pathological gambling.

The strength of our study was the use of random digit dialing of mobile phone users, which helped to increase the potential to generalize the findings to the general population of Thailand. However, a number of limitations should be considered in the interpretation of the study findings. Firstly, the lack of financial liquidity might have been perceived by some participants as being embarrassing to report, and social desirability could have influenced our study findings. Secondly, the relatively high refusal to participate could have introduced selection bias to our study findings. However, the demographics of the study participants resembled the demographics of the Thai population aged 18 years and over (51.8% female, mean age 45.8 years) (Thailand National Statistical Office, 2020), suggesting that non-participation might have happened at random and was unlikely to introduce selection bias.

## Conclusions

We assessed the extent that availability of emergency cash reserves in the amount of 5,000 Thai Bahts ($160 US) modified the association between economic distress and behavioral health outcomes during the COVID-19 pandemic in the general adult population of Thailand. Although there were no statistically significant additive or multiplicative effect modifications, study participants with emergency cash reserves had a consistently lower prevalence of anxiety, depressive symptoms, and history of gambling than participants without emergency cash reserves. The findings provide potentially useful basic information for stakeholders in behavioral health and economics. Further studies should consider qualitative data collection and longitudinal study design in order to explore these associations at greater depths.

## Supplemental Information

10.7717/peerj.13307/supp-1 Supplemental Information 1 R codes to replicate the analyses leading to the study findings. The directory of the file was redacted for security purposes.

10.7717/peerj.13307/supp-2 Supplemental Information 2 Data set with variables required to replicate the study findings.

10.7717/peerj.13307/supp-3 Supplemental Information 3 Data dictionary for the questionnaire and the data set.

10.7717/peerj.13307/supp-4 Supplemental Information 4 Questionnaire (in Thai) with English translation in the second half.

## Additional Information and Declarations

### Data Availability

The following information was supplied regarding data availability: The data set and the codes needed to carry out the analyses and produce the results, the original questionnaire (in Thai) with English translation, and the data dictionary (codebook) are available in the Supplemental Files.
