# Supplementary material for: Modification of the association between experience of economic distress during the COVID-19 pandemic and behavioral health outcomes by availability of emergency cash reserves: findings from a nationally-representative survey in Thailand
Source: PeerJ. 2022 Apr 20;10:e13307. doi: 10.7717/peerj.13307 (PMC9034704; doi:10.7717/peerj.13307)
Supplement: Supplemental Information 4 [file peerj-10-13307-s004.pdf]

ชื่อผู้ตอบ.. เบอร์โทรฯ...

สคริปต์การให้ข้อมูลแก่ผู้ตอบ (Participant Information Script)

ศูนย์วิจัย SAB ร่วมกับศูนย์วิจัยปัญหาสุรา (ศวส.) ภายใต้การสนับสนุนของ สสส. ขอความกรุณาท่านตอบแบบสอบถามภายใต้โครงการ **"การสำรวจพฤติกรรมการดื่มเครื่องดื่มแอลกอฮอล์ ผลกระทบทางเศรษฐกิจ และพฤติกรรมสุขภาพในช่วงการแพร่ระบาดของไวรัสโควิด-19"** โดยใช้เวลาประมาณ 5 นาที ครับ/ค่ะ

เนื่องจากโครงการนี้มีเป้าหมายเป็นประชาชนอายุ 18 ปีขึ้นไปเท่านั้น จึงขอรบกวนถามว่าท่านอายุเท่าใด? __________ ปี

[ ] อายุ 18 ปีขึ้นไป (แจ้งข้อมูลต่อไป) [ ] อายุต่ำกว่า 18 ปี **(ยุติการแจ้งข้อมูล กล่าวขอบคุณ และปิดการสัมภาษณ์)**

ระหว่างตอบแบบสอบถามนี้ ท่านสามารถปฏิเสธการตอบคำถามข้อใดก็ได้ สามารถขอหยุดการสัมภาษณ์ได้ในทันที และการตอบแบบสอบถามนี้ไม่มีค่าตอบแทนให้ท่าน ในการรายงานผล ศูนย์วิจัย SAB และศูนย์วิจัยปัญหาสุราจะวิเคราะห์และนำเสนอผลการสำรวจเป็นภาพรวม และกระผม/ดิฉัน จะไม่บันทึกชื่อหรือเบอร์โทรศัพท์ของท่านในข้อมูลสำรวจ หากท่านมีคำถามเพิ่มเติมเกี่ยวกับโครงการนี้ ท่านสามารถติดต่อ ดร.สุริยัน บุญแท้ ผู้จัดการโครงการ หรือคุณจำปา จันทร์ตรา ผู้ประสานงานโครงการ โทร. 02 941 1757

"ท่านมีคำถามอะไรเกี่ยวกับโครงการสำรวจฯ นี้หรือไม่?" (ผู้สัมภาษณ์ตอบคำถามของอาสาสมัคร)

สคริปต์การให้ความยินยอมทางวาจา (Verbal Informed Consent Script)

"ท่านยินยอมให้สัมภาษณ์ในโครงการสำรวจฯ นี้หรือไม่?"

[ ] ยินยอม [ ] ไม่ยินยอมให้สัมภาษณ์ในโครงการฯ

(ในกรณีที่ไม่ยินยอม) "ขอบคุณที่สละเวลา และขออภัยในความไม่สะดวกครับ/ค่ะ" (วางสาย)

(บันทึกหางเสียง (คำลงท้ายประโยคที่ผู้ตอบใช้) [ ] ครับ [ ] ค่ะ [ ] อื่นๆ

(ในกรณีที่ยินยอม)

"ในอีก 1-2 เดือนข้างหน้า ทางศูนย์วิจัย SAB อาจมีการเก็บข้อมูลต่อเนื่องเพื่อศึกษาแนวโน้มระยะยาวของผู้เข้าร่วมการสำรวจ ท่านยินยอมให้กระผม/ดิฉัน ติดต่อท่านอีกหรือไม่"

[ ] ยินยอม [ ] ไม่ยินยอม ให้ติดต่อซ้ำ

ข้าพเจ้าขอรับรองว่าได้ชี้แจงข้อมูลแก่ผู้ตอบ และขอความยินยอมทางวาจาตามสคริปต์นี้จริง

ลงนาม ________________________ ผู้สัมภาษณ์ วัน เดือน ปี _________________________

คำถามสัมภาษณ์

หมวดที่ 1 การดื่มแอลกอฮอล์

เครื่องดื่มแอลกอฮอล์ หมายถึง เครื่องดื่มทุกประเภทที่มีส่วนผสมของแอลกอฮอล์ เช่น เหล้า เบียร์ ไวน์ สุราพื้นบ้านต่างๆ

1. <u>*ในชีวิตนี้*</u> ท่านเคยดื่มสุราหรือเครื่องดื่มแอลกอฮอล์หรือไม่ (ไม่นับรวมจิบเพียงเล็กน้อย)

[ ] 0. ไม่เคยดื่มเลยในชีวิตนี้ **(ข้ามไปตอบข้อ 10.)** [ ] 1. เคยดื่ม [ ] 9. ไม่ตอบ **(ข้ามไปตอบข้อ 10.)**

2. <u>*หากท่านเคยดื่ม*</u> ท่านได้ดื่มสุราหรือเครื่องดื่มแอลกอฮอล์<u>*ในช่วง 12 เดือนที่ผ่านมา*</u>หรือไม่

[ ] 0. ไม่ได้ดื่ม **(ข้ามไปตอบข้อ 10.)** [ ] 1. ดื่ม [ ] 9. ไม่ตอบ **(ข้ามไปตอบข้อ 10.)**

3. <u>*โดยทั่วไป*</u> ท่านดื่มบ่อยแค่ไหน<u>*ในช่วง 12 เดือนที่ผ่านมา*</u>

[ ] 1. นานๆ ครั้ง (1-3 วัน/ปี) [ ] 2. นานๆ ครั้ง (4-7 วัน/ปี) [ ] 3. นานๆ ครั้ง (8-11 วัน/ปี)
[ ] 4. ทุกเดือน (1-3 วัน/เดือน) [ ] 5. ทุกสัปดาห์ (1-2 วัน/สัปดาห์) [ ] 6. วันเว้นวัน (3-4 วัน/สัปดาห์)
[ ] 7. เกือบทุกวัน (5-6 วัน/สัปดาห์) [ ] 8. ทุกวัน (7 วัน/สัปดาห์) [ ] 9. ไม่ตอบ

4. <u>*ในช่วง 12 เดือนที่ผ่านมา*</u> ท่านดื่มที่ไหนบ่อยครั้งที่สุด

[ ] 1. บ้านตนเอง [ ] 2. บ้านคนอื่น [ ] 3. ร้านอาหาร [ ] 4. ผับ บาร์ คาราโอเกะ
[ ] 5. งานเลี้ยง เช่น งานแต่งงาน งานศพ งานสโมสร [ ] 6. งานบุญประเพณีวัฒนธรรม
[ ] 7. งานรื่นเริงที่เอกชนจัด เช่น งานคอนเสิร์ต [ ] 8. หน้าร้านขายของชำ/ ร้านสะดวกซื้อ
[ ] 9. ศาลา/ ที่นั่งริมทาง/ ที่สาธารณะในชุมชน [ ] 10. อื่นๆ ระบุ .... [ ] 99. ไม่ตอบ

5. <u>*ในช่วง 12 เดือนที่ผ่านมา*</u> ท่านเคยดื่มหนักหรือดื่มปริมาณมากในครั้งเดียวหรือไม่

**เกณฑ์การพิจารณา "ดื่มมาก/ดื่มหนัก"** ได้แก่	สุราขาว/เชี่ยงชุน/ยาดองเหล้า: 5 เป็ก หรือ 1/4 ขวดใหญ่ หรือ 1/5 ขวดกลาง
	สุรากลั่น/ผสม: 1/4 ขวดใหญ่ หรือดื่มเพียว 5 เป็ก หรือ 8 แก้วผสม
	เบียร์: 4 กระป๋อง หรือ 2 ขวดใหญ่
	ไวน์/แชมเปญ: 1 ขวดใหญ่หรือ 4 แก้วไวน์
	น้ำผลไม้ผสมแอลกอฮอล์/ไวน์คูลเลอร์: 4.5 ขวด หรือ 4.5 กระป๋อง
	เหล้าหมัก (กระแช่/สาโท/อุ/สุราพื้นเมือง): 1 ขวดใหญ่ หรือ 2 แก้วครึ่ง

[ ] 1. ไม่เคยดื่มหนัก **(ข้ามไปตอบข้อ 7.)** [ ] 2. เคยดื่มหนัก [ ] 9. ไม่ตอบ **(ข้ามไปตอบข้อ 7.)**

6. <u>หากเคยดื่มมาก/ดื่มหนัก</u> ท่านดื่มมาก/ดื่มหนักบ่อยแค่ไหน<u>*ในช่วง 12 เดือนที่ผ่านมา*</u>

[ ] 1. นานๆ ครั้ง (1-3 วัน/ปี) [ ] 2. นานๆ ครั้ง (4-7 วัน/ปี) [ ] 3. นานๆ ครั้ง (8-11 วัน/ปี)
[ ] 4. ทุกเดือน (1-3 วัน/เดือน) [ ] 5. ทุกสัปดาห์ (1-2 วัน/สัปดาห์) [ ] 6. วันเว้นวัน (3-4 วัน/สัปดาห์)
[ ] 7. เกือบทุกวัน (5-6 วัน/สัปดาห์) [ ] 8. ทุกวัน (7 วัน/สัปดาห์) [ ] 9. ไม่ตอบ

7. ท่านได้ดื่มสุราหรือเครื่องดื่มแอลกอฮอล์<u>*ในช่วง 30 วันที่ผ่านมา*</u>หรือไม่ หากดื่ม เทียบกับช่วงก่อนการระบาดของโควิดแล้วดื่มน้อยลงหรือเพิ่มขึ้น

[ ] 1. ไม่ได้ดื่ม (ระบุเหตุผลแล้วข้ามไปตอบข้อ 10.) **[ ] 2. ดื่มน้อยลง**

☞ <u>กรณีไม่ได้ดื่ม/ ดื่มน้อยลง</u> เป็นเพราะอะไร *(ตอบได้มากกว่า 1 ข้อ)*

( ) 1. ไม่ได้พบปะสังสรรค์ ( ) 2. ต้องการรักษาสุขภาพเพราะกลัวติดโควิด ( ) 3. งานยุ่ง/ ไม่มีเวลา ( ) 4. ไม่มีเครื่องดื่มฯ เก็บไว้
( ) 5. ปกติก็ไม่ค่อยดื่มอยู่แล้ว ( ) 6. ไม่สบายจึงหยุดดื่ม ( ) 7. ต้องการประหยัดเงิน ( ) 8. ไม่มีเหตุผล

[ ] 3. ดื่มเท่าเดิม **[ ] 4. ดื่มเพิ่มขึ้น** **[ ] 9. ไม่ตอบ (ข้ามไปตอบข้อ 10.)**

☞ <u>กรณีดื่มเท่าเดิม/ ดื่มเพิ่มขึ้น</u> เป็นเพราะอะไร *(ตอบได้มากกว่า 1 ข้อ)*

( ) 1. ดื่มเป็นประจำ/ ดื่มจนติด (อดไม่ได้) ( ) 2. มีคนชวนดื่ม ขัดไม่ได้ ( ) 3. ปกติก็ไม่ค่อยดื่มอยู่แล้ว
( ) 4. มีเวลาว่างมากขึ้น ( ) 5. เครียด/ เบื่อหน่าย/ เซ็ง ( ) 6. เหงาเพราะต้องอยู่คนเดียว/ แยกตัวอยู่บ้าน
( ) 7. มีเครื่องดื่มฯ เก็บไว้เยอะ ( ) 8. มีการพบปะสังสรรค์/ ตั้งวง/ ปาร์ตี้ดื่ม (ทั้งออนไลน์หรือวงจริง) ( ) 9. ไม่มีเหตุผล

8. *<u>ในช่วง 30 วันที่ผ่านมา</u>* ท่านดื่มที่ไหนบ่อยครั้งที่สุด

[ ] 1. บ้านตนเอง [ ] 2. บ้านคนอื่น [ ] 3. ร้านอาหาร [ ] 4. ผับ บาร์ คาราโอเกะ

[ ] 5. งานเลี้ยง เช่น งานแต่งงาน งานศพ งานสโมสร [ ] 6. งานบุญประเพณีวัฒนธรรม

[ ] 7. งานรื่นเริงที่เอกชนจัด เช่น งานคอนเสิร์ต [ ] 8. หน้าร้านขายของชำ/ ร้านสะดวกซื้อ

[ ] 9. ศาลา/ ที่นั่งริมทาง/ ที่สาธารณะในชุมชน [ ] 10. อื่นๆ ระบุ .... [ ] 99. ไม่ตอบ

9. *<u>ในช่วง 30 วันที่ผ่านมา</u>* ท่านเคยดื่มหนักหรือดื่มปริมาณมากในครั้งเดียวหรือไม่

[ ] 1. ไม่เคยดื่มหนัก [ ] 2. เคยดื่มหนัก [ ] 9. ไม่ตอบ

หมวดที่ 2 ผลกระทบทางเศรษฐกิจ และพฤติกรรมสุขภาพ

10. นับตั้งแต่รัฐบาลประกาศให้การระบาดของโรคโควิด-19 เป็นสถานการณ์ฉุกเฉิน ท่านประสบปัญหาเหล่านี้หรือไม่ <u>หากเคย</u> ในช่วง 30 วันที่ผ่านมา ท่านยังประสบปัญหานั้นอยู่หรือไม่ (นับรวมทั้งกรณีเป็นผลต่อเนื่อง และการเกิดซ้ำ)

**ปัญหา**	**นับตั้งแต่รัฐบาลประกาศสถานการณ์ฉุกเฉิน** (หากตอบ “ไม่เคย”/ “ไม่ตอบ” ให้ไปข้อถัดไป)	(เฉพาะ “เคย”) **ในช่วง 30 วันที่ผ่านมา** ประสบปัญหานั้นอยู่หรือไม่
1) ถูกเลิกจ้าง หรือสูญเสียแหล่งรายได้หลักเป็นเวลาสามเดือนขึ้นไป	[ ] 1. เคย [ ] 2. ไม่เคย [ ] 9. ไม่ตอบ	[ ] 1. มีปัญหา [ ] 2. ไม่มี [ ] 9. ไม่ตอบ
2) ถูกลดเงินเดือน/วันทำงาน	[ ] 1. เคย [ ] 2. ไม่เคย [ ] 9. ไม่ตอบ	[ ] 1. มีปัญหา [ ] 2. ไม่มี [ ] 9. ไม่ตอบ
3) ไม่สามารถจ่ายค่าไฟ ค่าน้ำ หรือค่าโทรศัพท์ได้ตามกำหนด	[ ] 1. เคย [ ] 2. ไม่เคย [ ] 9. ไม่ตอบ	[ ] 1. มีปัญหา [ ] 2. ไม่มี [ ] 9. ไม่ตอบ
4) ไม่สามารถผ่อนบ้าน/ ผ่อนรถ/ ค่างวดหรือจ่ายค่าเช่าได้ตามกำหนด	[ ] 1. เคย [ ] 2. ไม่เคย [ ] 9. ไม่ตอบ	[ ] 1. มีปัญหา [ ] 2. ไม่มี [ ] 9. ไม่ตอบ
5) ต้องจำนำหรือขายของเพื่อให้ได้เงินสดมาใช้จ่ายในเรื่องจำเป็น	[ ] 1. เคย [ ] 2. ไม่เคย [ ] 9. ไม่ตอบ	[ ] 1. มีปัญหา [ ] 2. ไม่มี [ ] 9. ไม่ตอบ
6) เคยอดข้าวอย่างน้อยหนึ่งมื้อ	[ ] 1. เคย [ ] 2. ไม่เคย [ ] 9. ไม่ตอบ	[ ] 1. มีปัญหา [ ] 2. ไม่มี [ ] 9. ไม่ตอบ
7) ขอความช่วยเหลือทางการเงินจากเพื่อนหรือญาติ	[ ] 1. เคย [ ] 2. ไม่เคย [ ] 9. ไม่ตอบ	[ ] 1. มีปัญหา [ ] 2. ไม่มี [ ] 9. ไม่ตอบ
8) ขอความช่วยเหลือจากหน่วยงานสวัสดิการสังคม หรือมูลนิธิต่างๆ	[ ] 1. เคย [ ] 2. ไม่เคย [ ] 9. ไม่ตอบ	[ ] 1. มีปัญหา [ ] 2. ไม่มี [ ] 9. ไม่ตอบ

11. สมมุติว่าท่านมี<u>ค่าใช้จ่ายฉุกเฉิน</u>ประมาณ 5,000 บาท ท่านจะหาเงินมาจ่ายในส่วนนี้*<u>ภายในหนึ่งสัปดาห์</u>*ได้อย่างไร (รอฟังคำตอบ ตอบได้มากกว่าหนึ่งข้อ)

[ ] 1) ใช้บัตรเครดิต และชำระหนี้เต็มจำนวนในรอบหน้า [ ] 2) ใช้บัตรเครดิต และทยอยชำระเป็นงวดๆ

[ ] 3) ใช้เงินในบัญชีเงินฝากออมทรัพย์ ใช้เงินสด หรือใช้สินทรัพย์สภาพคล่องอื่นๆ (เช่น ขายคืนกองทุนรวมเป็นเงินสด)

[ ] 4) ใช้เงินกู้ธนาคาร หรือสินเชื่อ [ ] 5) ยืมเพื่อนหรือญาติ

[ ] 6) ใช้เงินกู้นอกระบบ เบิกเงินล่วงหน้า หรือถอนเงินเกินจำนวน [ ] 7) เอาของส่วนตัวไปขาย [ ] 8) จำนำทรัพย์สิน

[ ] 9) ไม่สามารถหาเงินจัดการค่าใช้จ่ายดังกล่าวได้ [ ] 10) อื่นๆ ระบุ .... [ ] 99) ไม่ตอบ

12. หากท่าน*<u>สูญเสียแหล่งรายได้หลัก</u>* (เช่น งานที่ทำ สวัสดิการ เงินช่วยเหลือจากครอบครัว) *<u>เป็นเวลา 3 เดือน</u>* ท่านจะจัดการค่าใช้จ่ายต่างๆ ของท่านอย่างไร (รอฟังคำตอบ ตอบได้มากกว่าหนึ่งข้อ)

[ ] 1) ใช้บัตรเครดิต และชำระหนี้เต็มจำนวนในรอบหน้า [ ] 2) ใช้บัตรเครดิต และทยอยชำระเป็นงวดๆ

[ ] 3) ใช้เงินในบัญชีเงินฝากออมทรัพย์ ใช้เงินสด หรือใช้สินทรัพย์สภาพคล่องอื่นๆ (เช่น ขายคืนกองทุนรวมเป็นเงินสด)

[ ] 4) ใช้เงินกู้ธนาคาร หรือสินเชื่อ [ ] 5) ยืมเพื่อนหรือญาติ

[ ] 6) ใช้เงินกู้นอกระบบ เบิกเงินล่วงหน้า หรือถอนเงินเกินจำนวน [ ] 7) เอาของส่วนตัวไปขาย [ ] 8) จำนำทรัพย์สิน

[ ] 9) ไม่สามารถหาเงินจัดการค่าใช้จ่ายดังกล่าวได้ [ ] 10) อื่นๆ ระบุ .... [ ] 99) ไม่ตอบ

13. *ในช่วง 2 สัปดาห์ที่ผ่านมา* ท่านถูกรบกวนด้วยปัญหาต่อไปนี้บ่อยเพียงใด

การถูกรบกวน	0. ไม่เลย	1. บางวัน (1-7 วัน)	2. เกินกว่า 7 วัน (8-11 วัน)	3. เกือบทุกวัน (12-14 วัน)
**แบบวัดความวิตกกังวล (GAD-7)**				
1) รู้สึกตึงเครียด วิตกกังวล หรือกระวนกระวาย				
2) ไม่สามารถหยุดหรือควบคุมความกังวลได้				
3) กังวลมากเกินไปในเรื่องต่างๆ				
4) ทำตัวให้ผ่อนคลายได้ยาก				
5) รู้สึกกระสับกระส่ายจนไม่สามารถนั่งนิ่งๆ ได้				
6) กลายเป็นคนขี้รำคาญ หรือ หงุดหงิดง่าย				
7) รู้สึกกลัวเหมือนว่าจะมีอะไรร้ายๆ เกิดขึ้น				
**แบบวัดอาการซึมเศร้า (PHQ-2)**				
1) รู้สึกเบื่อ ทำอะไรไม่เพลิดเพลิน				
2) รู้สึกหดหู่ เศร้า หรือท้อแท้สิ้นหวัง				

พฤติกรรมสุขภาพ

14. *นับรวมทั้งชีวิตที่ผ่านมา* คุณเคย*สูบบุหรี่หรือยาเส้น* รวมกันมากกว่า 5 ซอง หรือ 100 มวนหรือไม่

[ ] 1. ไม่เคยสูบบุหรี่เลย **(ข้ามไปตอบข้อ 16.)** [ ] 2. เคยสูบ แต่ทั้งชีวิตรวมไม่เกิน 5 ซอง หรือ 100 มวน

[ ] 3. เคย [ ] 9. ไม่ตอบ **(ข้ามไปตอบข้อ 16.)**

15. คุณสูบบุหรี่*ครั้งล่าสุด*เมื่อไร

[ ] 1. นานกว่า 12 เดือน [ ] 2. นานกว่า 30 วันที่ผ่านมา แต่ภายใน 12 เดือน

[ ] 3. นานกว่า 1 สัปดาห์ที่ผ่านมา แต่ภายใน 30 วัน [ ] 4. ภายใน 1 สัปดาห์ที่ผ่านมา [ ] 9. ไม่ตอบ

16. *ในช่วง 30 วันที่ผ่านมา* ในแต่ละสัปดาห์ คุณเล่นกีฬา/ออกกำลังกาย (ครั้งละ 20 นาทีขึ้นไป) บ่อยเพียงใด

[0] ไม่ออกเลย [1] 1-2 วันต่อสัปดาห์ [2] 3-4 วันต่อสัปดาห์ [3] 5-7 วันต่อสัปดาห์ [9] ไม่ตอบ

17. *ในช่วง 30 วันที่ผ่านมา* คุณนอนกลางคืน (หรือบางอาชีพเช่น รปภ. อาจนอนกลางวันแทนกลางคืน) โดยเฉลี่ยวันละกี่ชั่วโมง

[1] 4 ชม. หรือน้อยกว่า [2] 5 ชม. [3] 6 ชม. [4] 7 ชม. [5] 8 ชม. หรือมากกว่า

[8] ไม่ทราบ ไม่แน่ใจ [9] ไม่ตอบ

18. *ในช่วง 30 วันที่ผ่านมา* คุณเล่นการพนันบ้างหรือไม่ (นับรวมหวยใต้ดิน และสลากกินแบ่งรัฐบาล)

[ ] 0. ไม่เล่น [ ] 1. เล่น [ ] 9. ไม่ตอบ

19. *ในช่วง 30 วันที่ผ่านมา* คุณเล่นวีดีโอเกมกี่วัน (นับรวมทั้งเกมในคอมฯ เกมมือถือ หรือเครื่องเล่นเกมอิเล็กทรอนิกส์ต่างๆ)

[0] ไม่เล่นเลย **(ข้ามไปตอบข้อ 21.)** [1] 1-2 วัน [2] 3-5 วัน [3] 6-9 วัน

[4] 10-19 วัน [5] 20 วันขึ้นไป [9] ไม่ตอบ **(ข้ามไปตอบข้อ 21.)**

20. ในวันที่คุณเล่นวีดีโอเกม คุณใช้เวลาเล่นวีดีโอเกมกี่ชั่วโมง

[0] น้อยกว่า 1 ชม. [1] 1-2 ชม. [2] 3-4 ชม. [3] มากกว่า 4 ชม. [9] ไม่ตอบ

21. *ในช่วง 30 วันที่ผ่านมา* คุณเล่นหรือใช้โซเชียลมีเดีย (Social Media) กี่วัน (นับรวมทุกแพลตฟอร์มรวมกัน)

[0] ไม่ใช้เลย **(ข้ามไปตอบข้อมูลทั่วไป)** [1] 1-2 วัน [2] 3-5 วัน [3] 6-9 วัน

[4] 10-19 วัน [5] 20 วันขึ้นไป [9] ไม่ตอบ **(ข้ามไปตอบข้อมูลทั่วไป)**

22. ในวันที่คุณใช้โซเชียลมีเดีย คุณใช้เวลากับโซเชียลมีเดียวันละกี่ชั่วโมง

[0] น้อยกว่า 1 ชม. [1] 1-2 ชม. [2] 3-4 ชม. [3] มากกว่า 4 ชม. [9] ไม่ตอบ

<u>ข้อมูลทั่วไปของผู้ตอบแบบสอบถาม</u>

1. เพศ [ ] 1 ชาย [ ] 2 หญิง

2. อายุ.......................ปี

3. สถานภาพสมรส

[ ] 1. โสด [ ] 2. สมรสและมีบุตร [ ] 3. สมรสแต่ไม่มีบุตร [ ] 4. หม้าย/ หย่า/ แยกกันอยู่

4. ระดับการศึกษาชั้นสูงสุดที่สำเร็จมา

[ ] 1. ไม่เคยเรียน [ ] 2. ประถมศึกษา [ ] 3. ม.ต้น [ ] 4. ม.ปลาย [ ] 5. ปวช. [ ] 6. ปวส.

[ ] 7. อนุปริญญา [ ] 8. ปริญญาตรี [ ] 9. สูงกว่าปริญญาตรี

5. อาชีพประจำที่มีรายได้หลัก

[ ] 1. ข้าราชการ/ รัฐวิสาหกิจ [ ] 2. พนักงานบริษัทเอกชน [ ] 3. ค้าขายรายย่อย/ บริการรายย่อย

[ ] 4. ธุรกิจส่วนตัว/ ผู้ประกอบการ [ ] 5. ผู้ใช้แรงงาน/ รับจ้างทั่วไป [ ] 6. เกษียณอายุ/ พ่อบ้าน/ แม่บ้าน

[ ] 7. เกษตรกร/ ประมง [ ] 8. อาชีพอิสระ อาทิ ทนายความ สถาปนิก [ ] 9. นักเรียน/ นักศึกษา

[ ] 10. ว่างงาน [ ] 11. อื่นๆ ระบุ ....

6. รายได้ส่วนบุคคลเฉลี่ยต่อเดือน

[1] ไม่เกิน 5,000 บาท [2] 5,001 - 10,000 บาท [3] 10,001 - 20,000 บาท

[4] 20,001 - 30,000 บาท [5] 30,001 - 40,000 บาท [6] 40,001 - 50,000 บาท [7] มากกว่า 50,000 บาท

7. อาศัยอยู่เขต/ อำเภอ......................... จังหวัด.............................

8. อาศัยอยู่ในพื้นที่ [ ] 1. กรุงเทพมหานคร [ ] 2. ในเขตเทศบาล [ ] 3. นอกเขตเทศบาล

...ขอขอบคุณในความอนุเคราะห์ครับ/ค่ะ

Name of respondent... Telephone...

Participant Information Script

The SAB research center in collaboration with the Centre for Alcohol Studies under the support of the ThaiHealth would like to ask you to answer the questionnaire in the project "**Survey of Alcohol Consumption, Economic Impacts, and Health Behaviors during the COVID-19 Pandemic**". The interview will take approximately 5 minutes.

As the target group of this project only includes people age 18 years and older, please allow me to ask how old you are? __________ Years

[ ] Age 18 or over (continue) [ ] Age under 18 years **(Stop the information process, say thank you and finish the interview)**

During this interview, you can refuse to answer any question. You can stop the interview at any time. There is no remuneration for your participation. In reporting the survey results, SAB and the Centre for Alcohol Studies will only present aggregate data. I will not record your name or telephone number in the survey data set. If you have any additional question, please contact Dr. Suriyan Boontae, Project Manager, or Ms. Champa Chandra, Project Coordinator, Tel. 02 941 1757

"Do you have any question about this survey??" (Interviewer answers the respondent's question)

Verbal Informed Consent Script

"Do you consent to be interviewed in this survey?"

[ ] Consent [ ] Does not consent to participate

(If participant does not consent) "Thank you for your time and we apologize for the inconvenience" (hang up)

(record the particle (sentence ending used by the respondent)) [ ] *khrub* [ ] *kha* [ ] others

(If participant consents)

"In 1-2 months, SAB research center may conduct follow-up data collection to study the long-term trend among the participants. Will you allow me to conduct you again?"

[ ] Consent [ ] Does not consent to follow-up contact

I certify that I have informed the respondent and asked for verbal informed consent according to this script

Sign ________________________ Interviewer Day Month Year _________________________

Interview Questions

Section 1. Alcohol Consumption

Alcoholic beverage refers to all beverages that contain alcohol e.g., whiskey, beer, wine, traditional wines

1. *In this lifetime*, have you ever had whiskey or other alcoholic beverages (not including small sips)?

[ ] 0. Never in this lifetime **(Skip to Question 10)** [ ] 1. Yes [ ] 9. Refuse to answer **(Skip to Question 10)**

2. *If yes,* have you had any whiskey or alcoholic beverage *within the past 12 months*?

[ ] 0. No **(Skip to Question 10)** [ ] 1. Yes [ ] 9. Refuse to answer **(Skip to Question 10)**

3. *Generally*, how often did you drink *during the past 12 months*?

[ ] 1. Occasionally (1-3 days/year) [ ] 2. Occasionally (4-7 days/year) [ ] 3. Occasionally (8-11 days/year)

[ ] 4. Monthly (1-3 days/month) [ ] 5. Weekly (1-2 days/week) [ ] 6. Every other day (3-4 days/week)

[ ] 7. Almost daily (5-6 days/week) [ ] 8. Daily (7 days/week) [ ] 9. Refuse to answer

4. *During the past 12 months*, where did you drink most frequently?

[ ] 1. Own home [ ] 2. Others' home [ ] 3. Restaurant [ ] 4. Pubs/Bars/Karaoke

[ ] 5. Parties (weddings, funerals, social clubs) [ ] 6. Traditional festivities

[ ] 7. Private sector events (e.g., concerts) [ ] 8. In front of convenience stores

[ ] 9. Roadside rest areas / public places in communities [ ] 10. Others, specify ....

[ ] 99. Refuse to answer

5. *During the past 12 months*, have you ever drunk in large quantity on a single occasion?

Criteria for "binge/large quantity" drinking

White spirits/Xiangshun/Yadong: 5 shots, 1/4 large bottle, or 1/5 mid-sized bottle

Distilled/blended whiskey: 1/4 large bottle, 5 neat shots, or 8 glasses of mixed drinks

Beer: 4 cans or 2 large bottles

Wine/Champagne: 1 large bottle or 4 glasses

Fruit cocktail/wine cooler: 4.5 bottles or 4.5 cans

Fermented drinks (krachae, sato, ou, traditional wines): 1 large bottle or 2.5 glasses

[ ] 1. Never **(Skip to Question 7)** [ ] 2. Yes [ ] 9. Refuse to answer **(Skip to Question 7)**

6. If you have binge-drunk, how often did you binge-drink *during the past 12 months*?

[ ] 1. Occasionally (1-3 days/year) [ ] 2. Occasionally (4-7 days/year) [ ] 3. Occasionally (8-11 days/year)

[ ] 4. Monthly (1-3 days/month) [ ] 5. Weekly (1-2 days/week) [ ] 6. Every other day (3-4 days/week)

[ ] 7. Almost daily (5-6 days/week) [ ] 8. Daily (7 days/week) [ ] 9. Refuse to answer

7. Have you had any whiskey or alcoholic beverage *within the past 30 days*? If yes, did you drink more or less than prior to the COVID pandemic?

[ ] 1. No (Specify your reason and Skip to Question 10) **[ ] 2. Yes, drank less**

☞ If did not drink / drank less Why? *(more than 1 answers allowed)*

( ) 1. Did not have chance to socialize ( ) 2. need to maintain health from fear of COVID ( ) 3. Busy/No time

( ) 4. No beverage stashed ( ) 5. Generally did not drink anyways ( ) 6. I got sick, so I stopped

( ) 7. Need to save money ( ) 8. No reason

[ ] 3. Yes, drank the same **[ ] 4. Yes, drank more** **[ ] 9. Refuse to answer (Skip to Question 10)**

☞ <u>If drank the same / more</u> Why? *(more than 1 answers allowed)*

( ) 1. Habituation/Addiction (cannot go without) ( ) 2. Someone invited me, couldn't refuse

( ) 3. I normally did not drink ( ) 4. Have more free time ( ) 5. Stressed/Bored

( ) 6. Lonely because I had to isolate at home ( ) 7. I have lots of beverages stashed

( ) 8. There was a meetup / drinking circle / party (online or in real life) ( ) 9. No reason

8. *<u>During the past 30 days</u>*, where did you drink most frequently?

[ ] 1. Own home [ ] 2. Others' home [ ] 3. Restaurant [ ] 4. Pubs/Bars/Karaoke

[ ] 5. Parties (weddings, funerals, social clubs) [ ] 6. Traditional festivities

[ ] 7. Private sector events (e.g., concerts) [ ] 8. In front of convenience stores

[ ] 9. Roadside rest areas / public places in communities [ ] 10. Others, specify ....

[ ] 99. Refuse to answer

9. *<u>Within the past 30 days</u>*, have you ever drunk in large quantity on a single occasion?

[ ] 1. Never [ ] 2. Yes [ ] 9. Refuse to answer

Section 2. Economic Impacts and Health Behaviors

10. Since the government declared the state of emergency on the COVID-19 pandemic, have you experienced any of the following problems? <u>If yes</u>, within the past 30 days, have you experienced those problems (including continuation and recurrence)

Problem	**Since the state of emergency declaration** (if “No” or “Refuse to answer”, skip to the next problem)	**(Only if “Yes”) Have you experienced that problem in the past 30 days?**
1) Lost your work or main source of income for three months or longer	[ ] 1. Yes [ ] 2. No [ ] 9. Refuse	[ ] 1. Yes [ ] 2. No [ ] 9. Refuse
2) Had a reduction in pay/work hours	[ ] 1. Yes [ ] 2. No [ ] 9. Refuse	[ ] 1. Yes [ ] 2. No [ ] 9. Refuse
3) Could not pay electricity, water, or telephone bills on time	[ ] 1. Yes [ ] 2. No [ ] 9. Refuse	[ ] 1. Yes [ ] 2. No [ ] 9. Refuse
4) Could not pay the home mortage / car payment / installment / or rent on time	[ ] 1. Yes [ ] 2. No [ ] 9. Refuse	[ ] 1. Yes [ ] 2. No [ ] 9. Refuse
5) Pawned or sold something to raise the money for necessity	[ ] 1. Yes [ ] 2. No [ ] 9. Refuse	[ ] 1. Yes [ ] 2. No [ ] 9. Refuse
6) Went without meal on at least one occasion	[ ] 1. Yes [ ] 2. No [ ] 9. Refuse	[ ] 1. Yes [ ] 2. No [ ] 9. Refuse
7) Asked for financial help from friends or family	[ ] 1. Yes [ ] 2. No [ ] 9. Refuse	[ ] 1. Yes [ ] 2. No [ ] 9. Refuse
8) Asked for help from social service agencies or charities	[ ] 1. Yes [ ] 2. No [ ] 9. Refuse	[ ] 1. Yes [ ] 2. No [ ] 9. Refuse

11. Suppose that you have an emergency expense that costs 5,000 Bahts. How would you find money to pay for this expense within a week? (No prompting, wait for answer, more than one answers allowed)

[ ] 1) Put it on my credit card and pay it off in full at the next statement

[ ] 2) Put it on my credit card and pay it off over time

[ ] 3) With the money in savings account, cash, or other liquid assets (e.g., converting mutual funds to cash)

[ ] 4) Using money from a bank loan or line of credit [ ] 5) By borrowing from a friend or family member

[ ] 6) Using a payday loan, deposit advance, or overdraft [ ] 7) Selling my personal items

[ ] 8) Pawning my assets [ ] 9) I wouldn't be able to pay for the expense right now

[ ] 10) Other (please specify)____

[ ] 99) Refuse to answer

12. Suppose that you *lost your main source of income* (e.g., your job, welfare, financial aid from the family) *for a period of 3 months*. How would you find the money to manage your expenses? (No prompting, wait for answer, more than one answers allowed)

[ ] 1) Put it on my credit card and pay it off in full at the next statement

[ ] 2) Put it on my credit card and pay it off over time

[ ] 3) With the money in savings account, cash, or other liquid assets (e.g., converting mutual funds to cash)

[ ] 4) Using money from a bank loan or line of credit [ ] 5) By borrowing from a friend or family member

[ ] 6) Using a payday loan, deposit advance, or overdraft [ ] 7) Selling my personal items

[ ] 8) Pawning my assets [ ] 9) I wouldn't be able to pay for the expense right now

[ ] 10) Other (please specify)____

[ ] 99) Refuse to answer

13. *Over the last 2 weeks,* how often have you been bothered by the following problems?

**Problem**	0. Not at all	1. Several days (1-7 days)	2. More than 7 days (8-11 days)	3. Nearly every day (12-14 days)
**Generalised Anxiety Disorder Assessment (GAD-7)**				
1) Feeling nervous, anxious or on edge?				
2) Not being able to stop or control worrying?				
3) Worrying too much about different things?				
4) Trouble relaxing?				
5) Being so restless that it is hard to sit still?				
6) Becoming easily annoyed or irritable?				
7) Feeling afraid as if something awful might happen?				
**Depression Assessment Tool (PHQ-2)**				
1) Little interest or pleasure in doing things?				
2) Feeling down, depressed or hopeless?				

Health Behaviors

14. *In your entire lifetime*, have you smoked more than 5 packs or 100 sticks of *cigarettes or roll-your-own cigarettes*?

[ ] 1. Never **(Skip to Question 16)** [ ] 2. Yes, but less than 5 packs or 100 sticks in lifetime

[ ] 3. Yes [ ] 9. No **(Skip to Question 16)**

15. When was *the last time you smoked*?

[ ] 1. More than 12 months ago [ ] 2. More than 30 days ago but within past 12 months

[ ] 3. More than 1 week ago but within past 30 days [ ] 4. Within the past 1 week [ ] 9. Refuse to answer

16. *During the past 30 days*, how often did you play sports/exercise (more than 20 minutes per session)?

[0] None [1] 1-2 days/week [2] 3-4 days/week [3] 5-7 days/week [9] Refuse to answer

17. *During the past 30 days*, how long do you generally sleep at night (or during the day for some occupations such as security guard)?

[1] 4 hours or less [2] 5 hours [3] 6 hours [4] 7 hours [5] 8 hours or more

[8] Don't know / not sure [9] Refuse to answer

18. *Within the past* 30 days, have you gambled (including underground lottery and Thai state lottery)?

[ ] 0. No [ ] 1. Yes [ ] 9. Refuse to answer

19. *During the past 30 days*, on how many days did you play video games (Including PC games, mobile phone games, and other electronic gaming devices)?

[0] None **(Skip to Question 21)** [1] 1-2 days [2] 3-5 days [3] 6-9 days

[4] 10-19 days [5] 20 days or more [9] Refuse to answer **(Skip to Question 21)**

20. On the days that you played video games, how many hours did you play?

[0] Less than 1 hour [1] 1-2 hours [2] 3-4 hours

[3] More than 4 hours [9] Refuse to answer

21. *During the past 30 days*, on how many days did you use social media (all platforms combined)?

[0] None **(Skip to General Information)** [1] 1-2 days [2] 3-5 days [3] 6-9 days

[4] 10-19 days [5] 20 days or more [9] Refuse to answer **(Skip to General Information)**

22. On the days that you used social media, how many hours did you spend on social media?

[0] Less than 1 hour [1] 1-2 hours [2] 3-4 hours

[3] More than 4 hours [9] Refuse to answer

General Information about the Respondent

1. Sex [ ] 1 Male [ ] 2 Female

2. Age......................Years

3. Marital Status

[ ] 1. Single [ ] 2. Married with child(ren) [ ] 3. Married, no children [ ] 4. Widowed/Divorced/Separated

4. Highest level of education completed

[ ] 1. Never went to school [ ] 2. Primary school [ ] 3. Junior high school [ ] 4. High school [ ] 5. Vocational certificate [ ] 6. Vocational diploma

[ ] 7. Associate's degree [ ] 8. Bachelor's degree [ ] 9. Higher than bachelor's degree

5. Occupation for primary source of income

[ ] 1. Civil servant/state enterprise [ ] 2. Private sector employee

[ ] 3. Small-scale vendors/service providers [ ] 4. Business owner/entrepreneur

[ ] 5. Laborer/manual workers [ ] 6. Retired/homemaker [ ] 7. Agriculture/Fishery

[ ] 8. Independent professions e.g., lawyers, architects [ ] 9. Student [ ] 10. Unemployed

[   ] 11. Other. Please specify ....

6. Average monthly income

[1] No more than 5,000 Bahts   [2] 5,001 - 10,000 Bahts   [3] 10,001 - 20,000 Bahts

[4] 20,001 - 30,000 Bahts   [5] 30,001 - 40,000 Bahts   [6] 40,001 - 50,000 Bahts   [7] more than 50,000 Bahts

7. Residing in District .... Province ....

8. Residing in [   ] 1. Bangkok   [   ] 2. Within municipality area   [   ] 3. Outside municipality area

...*Thank you for your kind help*
